# Effective Connectivity of the Hippocampus Can Differentiate Patients with Schizophrenia from Healthy Controls: A Spectral DCM Approach

**DOI:** 10.1007/s10548-021-00868-8

**Published:** 2021-09-04

**Authors:** Lavinia Carmen Uscătescu, Lisa Kronbichler, Renate Stelzig-Schöler, Brandy-Gale Pearce, Sarah Said-Yürekli, Luise Antonia Reich, Stefanie Weber, Wolfgang Aichhorn, Martin Kronbichler

**Affiliations:** 1 Centre for Cognitive Neuroscience and Department of Psychology, University of Salzburg, Salzburg, Austria; 2 Neuroscience Institute, Christian-Doppler Medical Centre, Paracelsus Medical University, Salzburg, Austria; 3 Department of Psychiatry, Psychotherapy and Psychosomatics, Christian-Doppler Medical Centre, Paracelsus Medical University, Salzburg, Austria; 4 University Medical Center Hamburg – Eppendorf, Hamburg, Germany

**Keywords:** Schizophrenia, Hippocampus, Effective connectivity, Spectral dynamic causal modelling

## Abstract

We applied spectral dynamic causal modelling (Friston et al. in Neuroimage 94:396–407. 10.1016/j.neuroimage.2013.12.009, 2014) to analyze the effective connectivity differences between the nodes of three resting state networks (i.e. default mode network, salience network and dorsal attention network) in a dataset of 31 male healthy controls (HC) and 25 male patients with a diagnosis of schizophrenia (SZ). Patients showed increased directed connectivity from the left hippocampus (LHC) to the: dorsal anterior cingulate cortex (DACC), right anterior insula (RAI), left frontal eye fields and the bilateral inferior parietal sulcus (LIPS & RIPS), as well as increased connectivity from the right hippocampus (RHC) to the: bilateral anterior insula (LAI & RAI), right frontal eye fields and RIPS. In SZ, negative symptoms predicted the connectivity strengths from the LHC to: the DACC, the left inferior parietal sulcus (LIPAR) and the RHC, while positive symptoms predicted the connectivity strengths from the LHC to the LIPAR and from the RHC to the LHC. These results reinforce the crucial role of hippocampus dysconnectivity in SZ pathology and its potential as a biomarker of disease severity.

**Supplementary Information:**

The online version contains supplementary material available at 10.1007/s10548-021-00868-8.

## Introduction

Schizophrenia (SZ) is a common and debilitating psychiatric disorder, diagnosed in about 1% of the world’s population (Bhugra 2005). It is characterized by negative (e.g., social withdrawal and anhedonia) and positive symptoms (e.g., hallucinations and delusions). Given the wide range of deficits, clinicians have focused on identifying neuroimaging markers to aid in tracking disease progression and treatment response. Resting state networks (RSNs) are at the center of this endeavor (Parkes et al. 2020), and their activity appears to capture core deficits of SZ (e.g., Hudgens-Haney et al. 2020). Some RSNs, like the default mode network (DMN), increase their activity during rest and internally directed cognitive activity (Raichle 2015). The nodes belonging to the “core” DMN are the posterior cingulate cortex (PCC), the medial prefrontal cortex (MPFC), and the left and right inferior parietal cortex (LIPAR; RIPAR); in addition, the left and right hippocampus (LHC; RHC) are sometimes included (e.g., Ushakov et al. 2016), thus forming an “extended” DMN. Other networks, such as the salience network (SAN) and the dorsal attention network (DAN) show increased activity during externally directed cognitive processes (Uddin 2016; Vossel et al. 2014). The SAN comprises the dorsal anterior cingulate cortex (DACC) and the bilateral anterior insula (LAI; RAI). The DAN is composed of the bilateral frontal eye fields (LFEF; RFEF) and the bilateral inferior parietal sulcus (LIPS; RIPS).

Directed connectivity via stochastic or spectral dynamic causal modelling (spDCM) is the most recent approach to analyzing rsfMRI data (Friston et al. 2014) and holds great potential in revealing useful biomarkers in SZ. Compared to its stochastic counterpart, spDCM is more computationally efficient, and seems especially promising for clinical research as it has proven to be more sensitive to differentiating between patients and controls (Razi et al. 2015). So far, only a few spDCM studies involving SZ patients have been published (Chahine et al. 2017; Cui et al. 2015; Fang et al. 2018; Graña et al. 2017a, 2017b). Chahine et al. (2017) showed that directed connectivity within the left frontoparietal network correlates with the severity of negative symptoms and can capture similarities between SZ and first degree relatives. Cui et al. (2015) investigated the effective connectivity between nodes of the DMN (i.e., dorsal anterior cingulate cortex/DACC, medial prefrontal cortex/MPFC, and dorsolateral prefrontal cortex/DLPFC) and the hippocampus. They showed that SZ displayed increased directed connectivity from frontal nodes towards the left hippocampus, but decreased connectivity from the bilateral hippocampus towards frontal nodes. Fang et al. (2018) investigated effective connectivity among frontal nodes (i.e., DACC, DLPFC, and ventrolateral prefrontal cortex/VLPFC) and found overall decreased connectivity in first-episode SZ compared to HC. Additionally, this decreased connectivity seemed to be indicative of working memory impairments in SZ. Finally, Graña et al. (2017a, b) used spDCM and machine learning to validate previously proposed neural networks that might be involved in auditory hallucinations in SZ. Taken together, these examples support the unique insight that DCM analyses can offer in revealing clinically relevant biomarkers.

We analyzed directed connectivity between the three RSNs (i.e., DMN, SAN and DAN), as well as between their individual nodes. Of particular interest to us was the bilateral hippocampus, which was previously reported to show consistent differences between SZ and HC, both structurally (e.g., reduced gray matter volume in SZ) and functionally. We were therefore interested in assessing the directed connectivity group differences between the bilateral hippocampus and the other nodes of the three RSNs mentioned above.

To the best of our knowledge, there has only been one previous study assessing directed hippocampal dysconnectivity in SZ using spDCM (Cui et al. 2015). In a sample of first episode SZ, these authors found increased connectivity from the left DLPFC to the LHC, but decreased connectivity from the right ACC to the RHC and from the LHC to the DLPFC. Previous stochastic DCM research in SZ samples also showed that the hippocampus is differentially connected to other brain areas during rest. For example, Li et al. (2017) found significantly decreased connectivity from the auditory cortex to the hippocampus in patients with auditory verbal hallucinations compared to healthy controls (HC). Additionally, Lefebvre et al. (2016) further provided evidence supporting the involvement of the connectivity from the hippocampus to the SAN as being linked to the onset of hallucinations. These findings therefore promote the notion that the hippocampus might be a core driving area of hallucinatory experiences (Kapur 2003; Lodge and Grace 2011; Winton-Brown et al. 2014; Modinos et al. 2015).

Research using DCM has further shown that hippocampus connectivity is not only altered in SZ patients, but also in unaffected first degree relatives and in persons at ultra-high risk (UHR) for psychosis, thus appearing to be a promising biomarker of psychosis proneness. For example, Xi et al. (2016) showed, using stochastic DCM, that unaffected 1^st^ degree relatives, compared to HC, displayed increased connectivity from the left ACC to the RHC, but decreased connectivity from the right ACC to the RHC. In a task-based fMRI DCM study, Winton-Brown et al. (2017) further showed that the dysconnectivity of the hippocampal–basal ganglia–midbrain network during reward, novelty, and aversion processing can differentiate UHR from HC.

The hippocampus, as a hub for memory processes (Battaglia et al. 2011; Bernal-Casas et al. 2013) and for the whole connectome (Mišić et al. 2014), can offer additional clues regarding the mechanism of cognitive dysfunctions in SZ. In a task-based DCM study investigating associative learning, Banyai et al. (2011) found increased intrinsic connectivity in SZ compared to HC from the PFC to the hippocampus and from the hippocampus to the inferior temporal and superior parietal, even after controlling for the learning rate. In a memory task-based DCM study, Benetti et al. (2009) showed increased connectivity from the right posterior hippocampus to the right inferior frontal gyrus in HC compared to both first episode SZ and UHR, although the latter two groups did not differ significantly. Behaviorally, SZ, but not UHR, showed impaired memory performance compared to HC. Taken together, these results support the notion that the dysfunctional prefrontal-hippocampal coupling underlies memory impairments in SZ, and, moreover, that it could be used as a reliable intermediate phenotype biomarker (Bächner and Meyer-Lindenberg 2017).

Volumetric brain changes are also frequently reported in SZ, but it is debatable whether this occurs due to natural disease progression, or due to pharmacological treatment. In a longitudinal study of first episode SZ patents, Ho et al. (2011) showed that long-term antipsychotic treatment correlated with reduced Gray matter volume (GMV), while illness severity had little impact over volumetric brain alterations. Given that all the SZ patients in our sample were medicated, we also analyzed the impact of medication on effective connectivity strengths.

In the present study, we employed spDCM to investigate the effective connectivity differences between SZ and HC. First, we looked at the resting-state effective connectivity between individual nodes belonging to the DMN, SAN, and DAN, and the relationship between symptom severity and connection strengths. Second, we analyzed group differences in the hierarchical structure of these three networks, similar to Nee and D’Esposito (2016) and Zhou et al. (2018a). Third, using voxel-based morphometry (VBM) we analyzed GMV group differences in all the nodes included in our spDCM and re-assessed group differences in connection strengths while controlling for GMV. Because we were especially interested in the role of the hippocampus as the site of differential connectivity between HC and SZ, we opted for an extended configuration of the DMN, including the bilateral hippocampus. Based on previous effective connectivity findings, we expected to find increased connectivity between the hippocampus and other frontal areas belonging to the DMN and SAN, and by employing spectral DCM we aimed to further reveal the direction of this increased connectivity. The relationship between the hippocampus and the nodes belonging to the DAN was largely investigated in an exploratory fashion, given that previous effective connectivity research has largely overlooked this network. The main reason for including it in our analysis was due to SZ being also characterized by attention deficits.

## Materials and Methods

### Participants

The current study relies on the same participant data as already described in Kronbichler et al. (2018). Accordingly, we examined 25 all-male patients who had received a formal ICD-10 diagnosis (confirmed before study participation by certified psychiatrists) in the SZ spectrum group (F20; N = 24) or the schizoaffective disorders spectrum group (F25; N = 1). Please note that three participants of Kronbichler et al. did not complete a resting state fMRI scan sequence, wherefore they were excluded from the present study. The patients were recruited at the Department of Psychiatry, Psychotherapy and Psychosomatics at the Christian-Doppler Clinic in Salzburg, Austria. At the time of scanning, patients were clinically stable and medicated (mean chlorpromazine equivalent = 476.63, calculated according to Gardner et al. (2010), using the R package “chlorpromazineR” developed by Brown et al. 2021). The SCIP = Screen for Cognitive Impairment in Psychiatry (Purdon 2005) was used to ensure that patients were within the typical range with respect to their cognitive abilities. Symptom severity, as assessed with the Positive and Negative Syndrome Scale (PANSS; Kay et al. 1987), was found to be mild. PANSS scores were used to compute two sets of subscales; one three factor approach, which is also the most frequently used (Kay et al. 1987), and an alternative, five factor solution, according to the meta-analysis by Shafer and Dazzi (2019). Two of the patients did not complete the PANSS assessment but did complete the resting state scanning session. Efforts were made to recruit age matched HC. All 31 controls were screened for mental and physical health (via a standardized anamnesis procedure) and were excluded if they reported a current or history of mental or neurological disorder or a family history of psychiatric disorders. More details about the recruitment and assessment of the participants included in the present study can be found in Kronbichler et al. (2018). Demographic data and clinical scores are presented in Table 1.

**Table 1:** Demographic data of patients with schizophrenia (SZ) and healthy controls (HC)

	Schizophrenia patients (n = 25)	Healthy controls (n = 31)	p value
Age (year)	26.05 (4.9)	25.73 (4.5)	0.828
SCIP	68.74 (13.3)	84.85 (6.2)	< 0.001
CPZ (mg)	476.63 (232.01)		
Duration of illness (year)	4.04 (4.9)		
3F PANSS+	12.92 (6.6)		
3F PANSS−	14.23 (7.8)		
5F PANSS+	13.87 (5.6)		
5F PANSS−	15.78 (7)		
5F PANSS disorganization	15.3 (5.17)		
5F PANSS affect	9.4 (2.95)		
5F PANSS resistance	17.7 (2.7)		

Two participants from the SZ group did not complete the Positive and Negative Syndrome Scale (PANSS) assessment. 3F PANSS+ reflects the severity of positive symptoms, while 3F PANSS- that of negative symptoms — both according to the three factor structure. The following five scores were calculated according to the PANSS five factor structure: 5f PANSS+, 5F PANSS−, 5F PANSS Disorganization, 5F PANSS Affect, and 5F PANSS Resistance. Information on illness duration was only available for 24 patients, while only 23 completed the PANSS

*SCIP* Screen for Cognitive Impairment in Psychiatry (Purdon 2005), *CPZ* Chlorpromazine medication equivalent in mg

### Data Acquisition and Preprocessing

Imaging data were acquired with a Siemens Magnetom Trio 3T scanner (Siemens AG, Erlangen, Germany) using a 32-channel head coil. Functional images were obtained with a T2*-weighted gradient echo EPI sequence (TR = 2.250 ms, TE = 30 ms, matrix = 64 mm × 64 mm, FOV = 192 mm, flip angle = 70°). A gradient echo field map (TR = 488 ms, TE 1 = 4.49 ms, TE 2 = 6.95 ms) and a high-resolution (1 mm × 1 mm × 1 mm) structural scan with a T1-weighted MPRAGE sequence were recorded from each participant. Six dummy scans and a total of 110 resting state volumes (TR = 2.250 ms, TE 1 = 4.49 ms, TE 2 = 6.95 ms) were acquired. During the resting state sequence, participants were instructed to keep their eyes open and look at a fixation cross on the screen, while letting their mind wander.

For preprocessing and statistical analysis, SPM12 software running in a MATLAB R2013a environment (Mathworks Inc., Natick, MA, USA), and additional functions from AFNI3 were used. Functional images were realigned, de-spiked (with the AFNI 3ddespike function), unwarped, and corrected for geometric distortions using the fieldmap of each participant, and slice-time corrected. The high resolution structural T1-weighted image of each participant was processed and normalized with the CAT12 toolbox using default settings; each structural image was segmented into gray matter, white matter and CSF and denoised, then each image was warped into MNI space by registering it to the DARTEL template provided by the CAT12 toolbox via the high-dimensional DARTEL registration algorithm. Based on these steps, a skull stripped version of each image in native space was created. To normalize functional images into MNI space, the functional images were co-registered to the skull stripped structural image and the parameters from the DARTEL registration were used to warp the functional images, which were resampled to 3 mm × 3 mm × 3 mm voxels and smoothed with a 6 mm FWHM Gaussian kernel. Finally, an ICA-based strategy for Automatic Removal of Motion Artifacts (ICA-AROMA) was applied for motion correction.

### Data Analysis

**Fig. 1:**
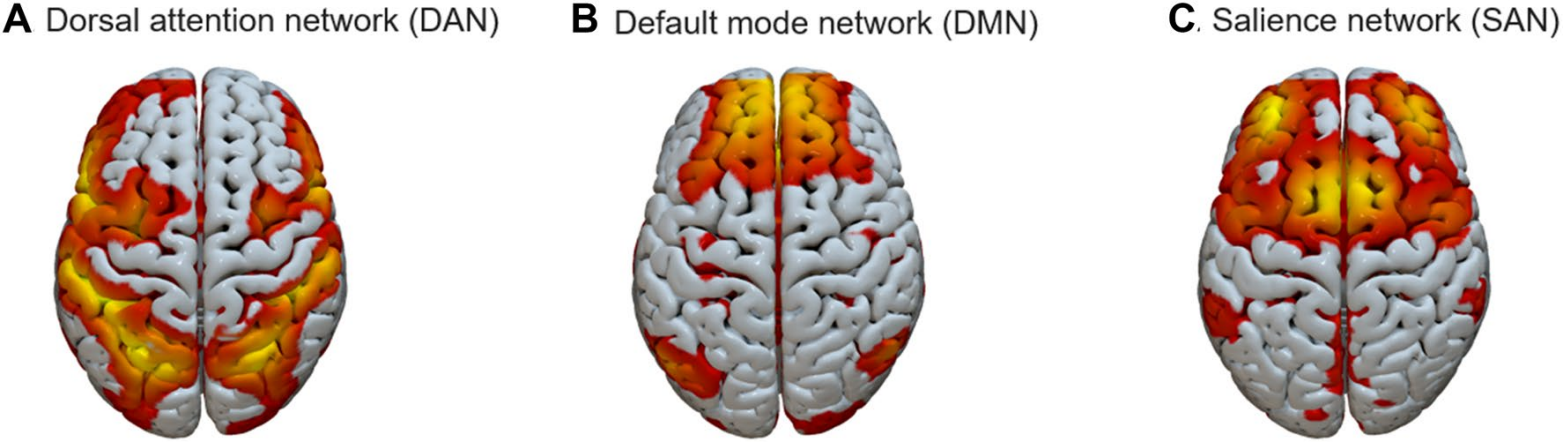
Spatial lay-out of the three resting state networks (RSNs): **A** Dorsal attention network (DAN); B Default mode network (DMN); **C** Salience network (SAN). Illustrations was created using the Surf Ice open- source toolbox (https://www.nitrc.org/projects/surfice/)

The three RSNs of interest to our current study were identified via spatial, constrained ICA, as implemented in the Group ICA for fMRI Toolbox (GIFT; https://trendscenter.org/software/gift/; Calhoun et al. 2001). The spatial templates used to constrain the ICA were those of Shirer et al. (2012), downloaded from http://findlab.stanford.edu/research. Group-level node coordinates are summarized in Table 2. These were obtained by averaging over individual node coordinates. The layout of the three networks, as mapped onto our sample data, can be seen in Fig. 1.

**Table 2:** Group-level node coordinates. Standard deviations are given in parentheses. The group-level coordinates were obtained by averaging across individual participants’ coordinates

Nodes	Mean MNI coordinates (SD)			Network
	x	y	z	
MPFC	− 0.64 (4.49)	52.13 (5.06)	12.08 (8.22)	DMN
LIPAR	− 44.59 (5.04)	− 64.66 (4.59)	29.71 (5.88)	DMN
RIPAR	48.73 (4.80)	− 58.92 (4.36)	28.60 (6.76)	DMN
PCC	− 1.03 (3.78)	− 52.08 (5.20)	25.91 (5.53)	DMN
LHC	− 23.38 (3.57)	− 29.27 (6.30)	− 13.18 (7.04)	DMN
RHC	25.48 (4.25)	− 21.32 (4.83)	− 16.39 (3.98)	DMN
DACC	0.32 (3.37)	18.97 (7.01)	41.65 (11.04)	SAN
LAI	− 42.26 (6.38)	15.84 (5.08)	− 3.41 (4.49)	SAN
RAI	44.41 (6.53)	15.98 (5.12)	− 2.80 (4.10)	SAN
LFEF	− 27.26 (3.98)	− 0.92 (4.96)	52.99 (4.42)	DAN
RFEF	29.17 (4.12)	− 1.06 (4.49)	54.07 (4.59)	DAN
LIPS	− 36.37 (8.42)	− 48.08 (11.71)	46.48 (4.86)	DAN
RIPS	34.57 (7.20)	− 50.48 (13.79)	47.57 (4.58)	DAN

*L* Left,*R* right, *PFC* prefrontal cortex, *IPAR* inferior parietal, *PCC* posterior cingulate cortex, *HC* hippocampus, *DACC* dorsal anterior cingulate cortex, *AI* anterior insula, *FEF* frontal eye fields, *IPS* inferior parietal sulcus, *DMN* default mode network, *SAN* salience network, *DAN* dorsal attention network

Following the identification of the ICA components corresponding to the DMN, DAN and SAN, we extracted individual time-courses for each node according to the following steps. First, group masks were created for each RSN, based on one-sample *t* tests of the respective component. This was followed by creating a within-subject design using the AROMA motion corrected preprocessed resting state files (Pruim et al. 2015), the single subject reconstructed time-courses for our three independent components, as well as covariates reflecting five principal components (PCs) of average signal for a white matter (WM) mask (based on each subject’s WM mask) and 5 PCs for cerebro-spinal fluid (CSF) mask (based on each subject’s ventricle mask) plus the 6 realignment parameters. Finally, individual time-courses for the DCM analysis were extracted from these designs by selecting the subjects’ local maximum from each ROI (constrained by the group mask for the respective ROI) for the contrast testing the association between the subject’s BOLD signal and the subjects ICA time-course. These local maxima were extracted as an 8 mm radius centered sphere.

Spectral DCM is a deterministic, linear alternative to stochastic DCM. Because the spectral generative model relies on second order statistics (i.e., cross spectra) of original time series, estimating their varying hidden states can be circumvented by estimating their time invariant covariance instead. This renders the model more computationally efficient, since the inversion scheme now only requires estimating the model’s parameters and hyper-parameters. A detailed mathematical treatment of spDCM can be found in Friston et al. (2014) and Razi et al. (2015).

The spDCM analysis was performed in SPM12 r7487 (Wellcome Trust Centre for Neuroimaging, London, UK; code available at: https://github.com/spm/spm12). At the first level, fully-connected models (i.e., between all nodes plus self-inhibitory connections) were estimated for each subject individually. At the second level analysis, the group model was built using the fully connected models from each subject, therefore capturing the between-subject effect on each of the modelled connections. Group differences in connection strength between the pre-defined nodes were assessed using a parametric empirical Bayes (PEB) model (Friston et al. 2015, 2016). The PEB is a hierarchical approach, in which the posterior density of model parameters is constrained by that of the previous level. Finally, post-hoc search (Rosa et al. 2012) was performed, which identifies and eliminates those parameters that do not contribute to model evidence.

Voxel based morphometry (VBM) was performed using the CAT12 toolbox (available at http://www.neuro.uni-jena.de/cat/). Gray matter volume of each node used in the spDCM analysis was extracted for each. All the other statistical analyses were performed in R 5.2 (R Core Team 2018).

## Results

### Effective/Directed Connectivity

**Fig. 2:**
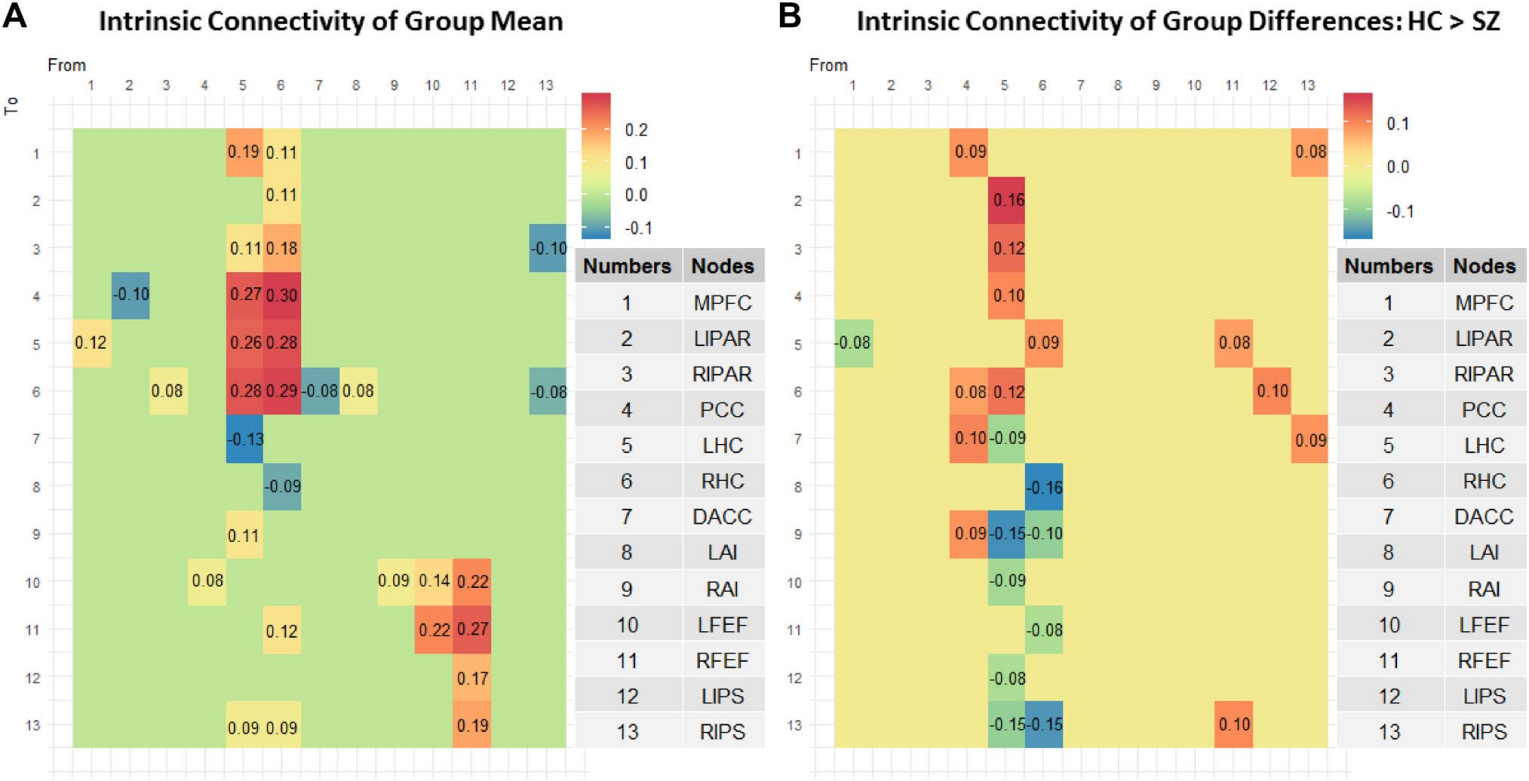
**A** Intrinsic connectivity matrix reflecting mean effective/directed connectivity between the 13 nodes across both groups. Only connections with a posterior probability > 0 0.95 are displayed; **B** Intrinsic connectivity matrix reflecting group differences (i.e., HC > SZ) in effective/directed connectivity between the 13 nodes. Only connections with a posterior probability > 0 0.95 are displayed. The results reflect connection strengths as a difference between those in the HC group and those in the SZ group; the color gradient therefore reflects positive values for those connections which were stronger in HC than in SZ, and negative values for those connections which were stronger in SZ compared to HC

**Fig. 3:**
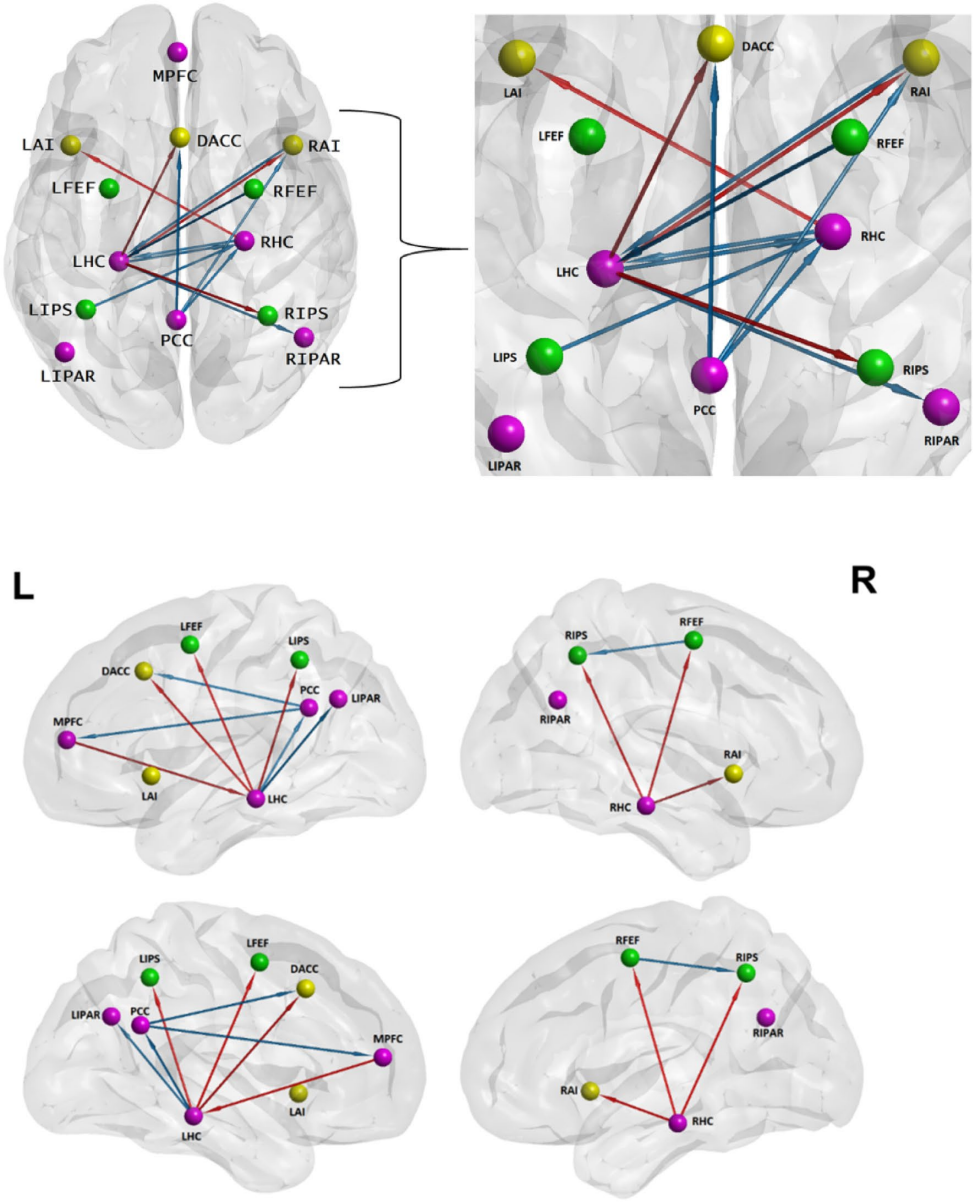
Top: Inter-hemispheric directed connections. Legend: orange arrows = SZ; blue arrows = HC; magenta nodes = DMN; green nodes = DAN; yellow nodes = SAN. Bottom: Intra-hemispheric directed connections. Legend: orange arrows = SZ; blue arrows = HC; magenta nodes = DMN; green nodes = DAN; yellow nodes = SAN

The connections with a posterior probability of more than 95% are depicted in Fig. 2 below. To ease comprehension of results, these connections are also mapped onto a brain template in Fig. 3.

BrainNet Viewer software (Xia et al. 2013; http://www.nitrc.org/projects/bnv/) was used to map the directed connections onto a brain surface template.

To quantify whether the LHC and RHC drive most of the significant connections, we modelled each column (i.e., out-going connections) from our connectivity matrices (Fig. 2A and B) as a binomial distribution with 13 possible outcomes, as there are 13 potential connections stemming from each node, including the self-connections. For each of the 13 possible outcomes, there is an equal likelihood of there being a connection present, hence a 0.5 probability for a connection being present or absent. Knowing what the number of significant connections stemming from each node is, we obtain that at the chance level, each node from our connectivity matrices can drive connections towards six other nodes. Therefore, any number of connections strictly lower than six would fall below chance level, while any number strictly higher than six and up to and including 13 would be above chance level. We can thus see that at the whole group level (Fig. 2A), it is the LHC and the RHC who drive an above chance level number of connections, with the LHC remaining dominant also when comparing HC to SZ (Fig. 2B). A mathematical treatment of the algorithm used for this analysis can be found in Loader et al. (2002).

**Fig. 4:**
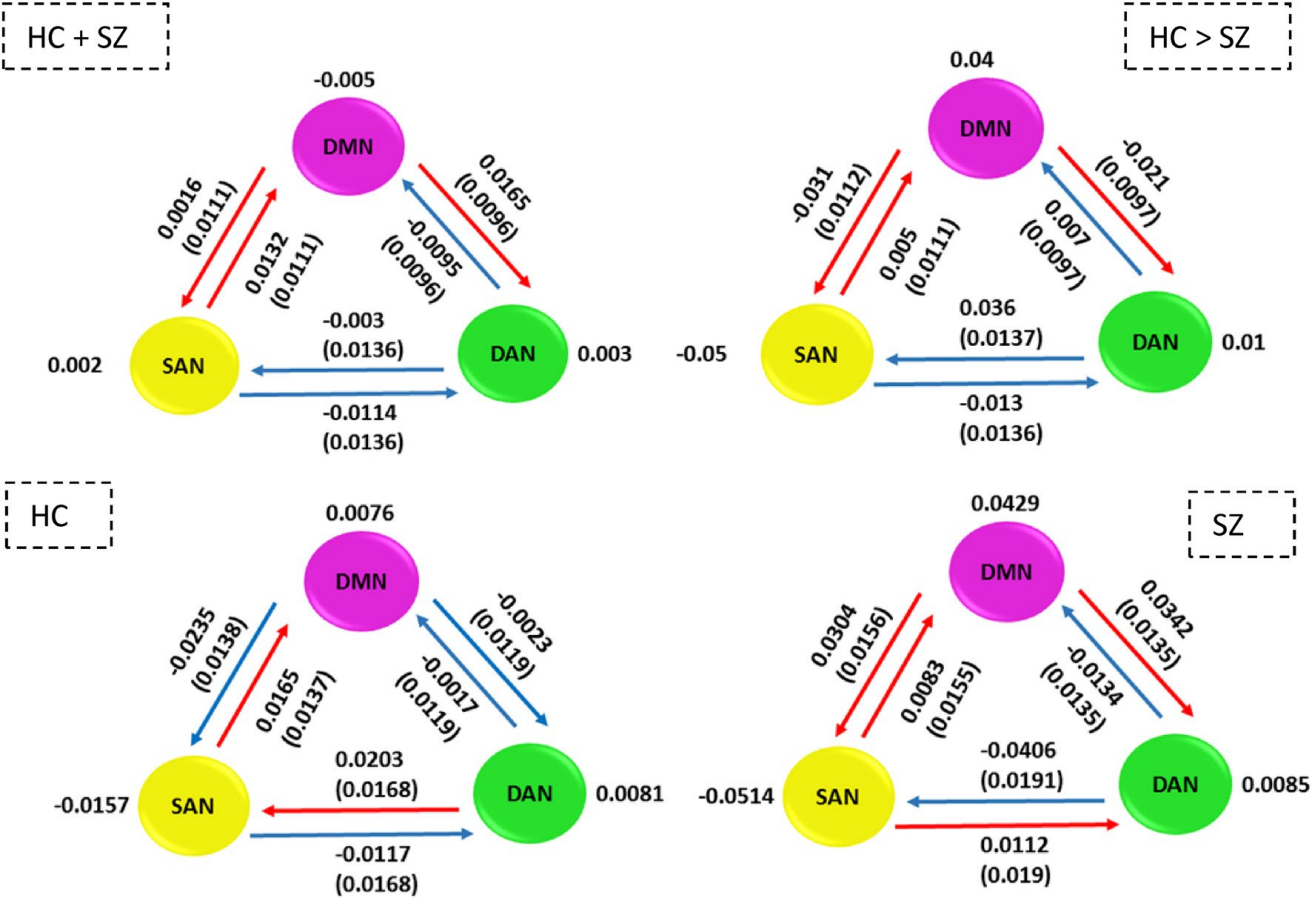
Hierarchical organization between the three RSNs, as well as individual hierarchical strengths. The uncertainty is reported in parentheses as standard deviation for each hierarchical connection. Blue arrows = inhibitory connections, red arrows = excitatory connections. The top left panel illustrates the hierarchical organization for the mean connectivity of the entire group (i.e., HC + SZ), while the top right one shows the hierarchical strengths for group differences (i.e., HC > SZ). The bottom left panel shows hierarchical strength for HC only, while the bottom right one for SZ only

To additionally quantify the hierarchical connection strength of our three RSNs, we computed, from the unthresholded mean group level connection strengths (Fig. 2A), the average connectivity strengths of each of our three RSNs, as well as between any given pair, bidirectionally (see Fig. 4). To achieve this, we followed the procedure described by Nee and D’Esposito (2016) and Zhou et al. (2018a). Additionally, like Zhou et al., we also computed the uncertainty of each between-network connection strength, which we report in parentheses, as standard deviations (Fig. 4). In the top left panel of Fig. 4, we illustrate hierarchical strengths computed on the mean connectivity strengths of the entire group mean (comprising both HC and SZ). The top right panel of Fig. 4 shows the hierarchical strengths computed on the group difference connectivity strengths (i.e., HC > SZ). In the bottom left panel of Fig. 4, the hierarchical strengths for HC are illustrated, while the hierarchical strengths for SZ are illustrated on the bottom right panel of Fig. 4.

In all four situations, the SAN excites the DMN, while the DAN inhibits the DMN. In HC it is the DAN which excites the SAN, whereas in SZ, this effect is reversed. Additionally, in HC, the DMN inhibits the DAN, but in SZ, it excites the DAN.

We further assessed the evidence strength of these findings by computing the posterior probability that the between-network connection strengths are different from zero. For the case of the two samples combined, depicted in the top-left of Fig. 4, the evidence for all the between-network connections was weak (≤ 73%). In the case of group differences depicted in the top-right of Fig. 4, very strong evidence was found for the increased excitatory influence from the DAN to the SAN in HC compared to SZ (> 99%), while weaker evidence was found for the rest of the hierarchical connections (≤ 85%). Strong evidence for increased excitatory connection from the DAN to the SAN in the SZ group was also found (> 96%), but weaker evidence (< 88%) for the other connections within the SZ and HC groups.

### The Relation Between Symptom Severity and the Strength of Directed Connectivity

We assessed the relation between connectivity strength and symptom severity (as reflected by the PANSS scores) within SZ group. Because in some cases we were concerned about the possibility of outlier points, but could not substantiate a decision to remove them, we decided to fit both simple linear and robust regression models. Symptom severity was computed according to both the three and the five PANSS factor solution.

#### PANSS 3 Factors: Negative Symptoms Severity and Connectivity Strength

**Fig. 5:**
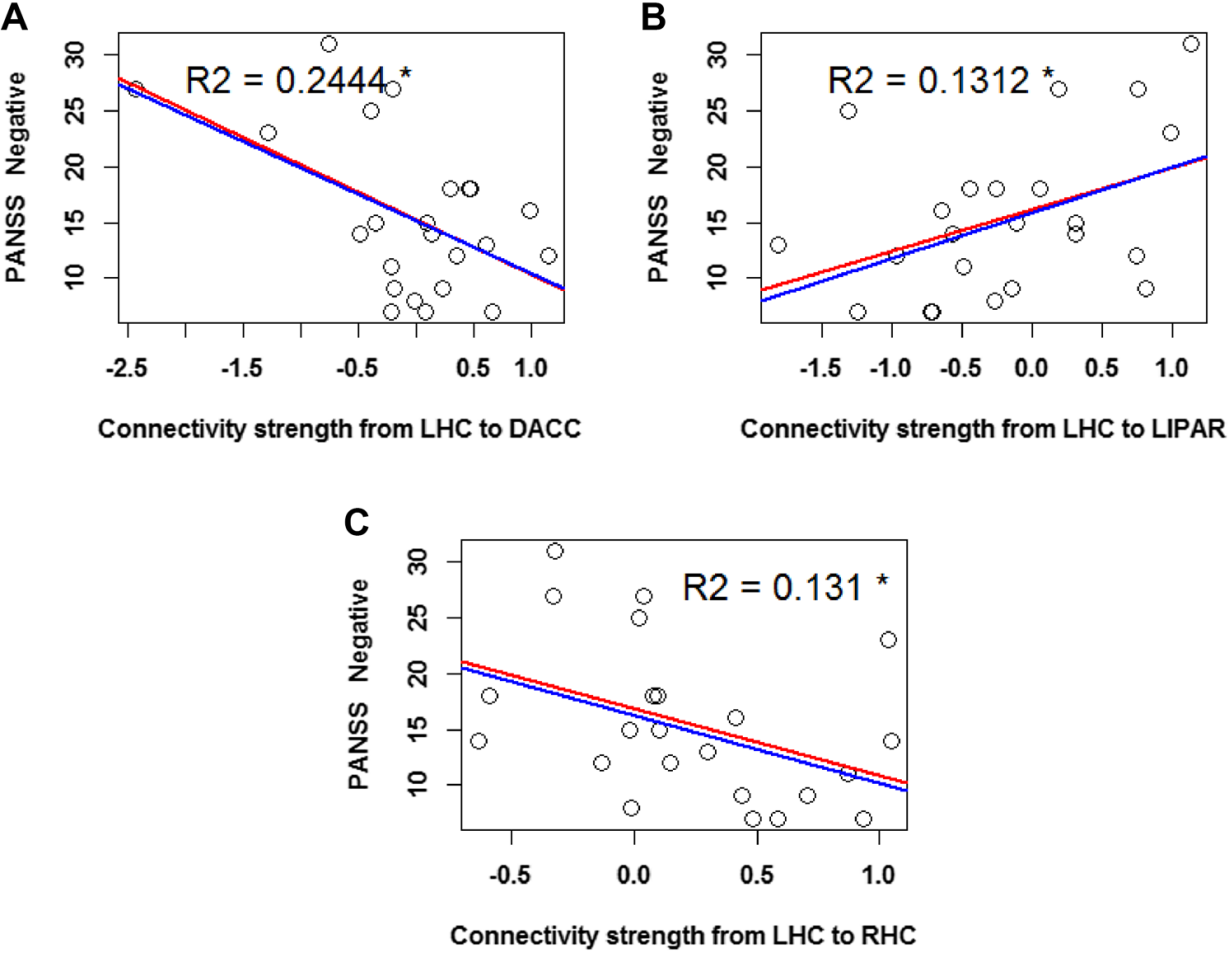
Linear regression with severity of negative symptoms (as reflected by PANSS 3 factor scores) as regressors. The predicted variables are the connectivity strengths of the following parameters: **A** from the LHC to the DACC **B** from the LHC to the LIPAR, and **C** from the LHC to the RHC. The red regression line reflects the fit of the simple linear regression model, while the blue line reflects that of the robust regression model. The plotted R^2^ values reflect the simple linear regression model fit. The asterisk indicates the uncorrected p < 0.05 significance level

A negative correlation *r* = − 0.53 between the severity of negative symptoms and connectivity strength from the LHC to the DACC was found, and a simple linear regression model (Fig. 5 A) further showed a significant (*p* = 0.009, *p*_*Bonferroni*_ = 0.23) relation between the two variables. The slope coefficient for the severity of negative symptoms was − 0.057, so connectivity strength decreases by 0.057 for every additional one unit increase of negative symptom severity. The adjusted R^2^ value was 0.2444, which indicates that 24.44% of the variation in LHC to DACC connectivity strength can be explained by negative symptom severity.

The severity of negative symptoms further positively correlated with the connectivity strength from the LHC to the LIPAR (*r* = 0.41), and a simple linear regression model (Fig. 5B) additionally showed a significant relation between the two variables (*p* = 0.05, *p*_*Bonferroni*_ = 1). The slope coefficient was 0.046, and the adjusted R^2^ value was 0.1312.

The severity of negative symptoms was also negatively correlated to the connectivity strength from the LHC to the RHC (*r* = − 0.41), and a simple linear regression model (Fig. 5C) additionally showed a significant relation between the two variables (*p* = 0.05, *p*_*Bonferroni*_ = 1). The model’s slope coefficient was − 0.028, and the adjusted R^2^ value was 0.131. Due to this connectivity parameter also significantly correlating with medication (see further down, under “Effects of illness duration and medication on directed connectivity strength in SZ”), we ran an additional partial correlation between the connection strength from the LHC to the RHS and the severity of negative symptoms, while controlling for medication. This correlation, while no longer significant (*p* = 0.07), remained non-negligible (r = − 0.39).

#### PANSS 3 Factors: Positive Symptoms Severity and Connectivity Strength

**Fig. 6:**
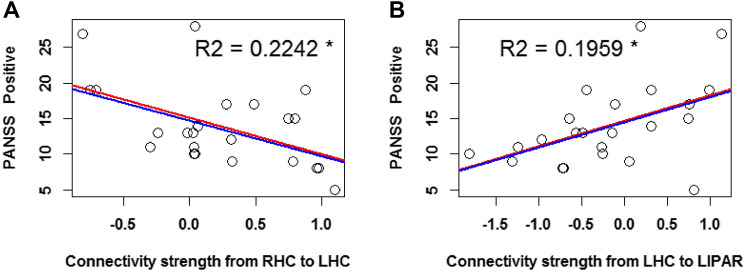
Linear regression with severity of positive symptoms (as reflected by PANSS 3 factor scores) as regressors. The predicted variables are the connectivity strengths of the following parameters: **A** from the RHC to the LHC, and **B** from the LHC to the LIPAR. The red regression line reflects the fit of the simple linear regression model, while the blue line reflects that of the robust regression model. The plotted R^2^ values reflect the simple linear regression model fit. The asterisk indicates an uncorrected p < 0.05 significance level

The severity of positive symptoms was negatively correlated to the connectivity strength from the RHC to the LHC (*r* = −0.51). A simple linear regression model (Fig. 6A) further showed that the relation between these variables was significant (*p* = 0.013, *p*_*Bonferroni*_ = 0.48), with a slope of − 0.05 and an adjusted R^2^ value of 0.2242.

Finally, the severity of positive symptoms was positively correlated to the connectivity strength from the LHC to the LIPAR (*r* = 0.48). A simple linear regression model (Fig. 6B) further showed that the relation between these variables was significant (*p* = 0.009, *p*_*Bonferroni*_ = 0.32), with a slope of 0.07 and an adjusted R^2^ value of 0.1959.

#### PANSS 5 Factors: Positive Symptoms Severity and Connectivity Strength

**Fig. 7:**
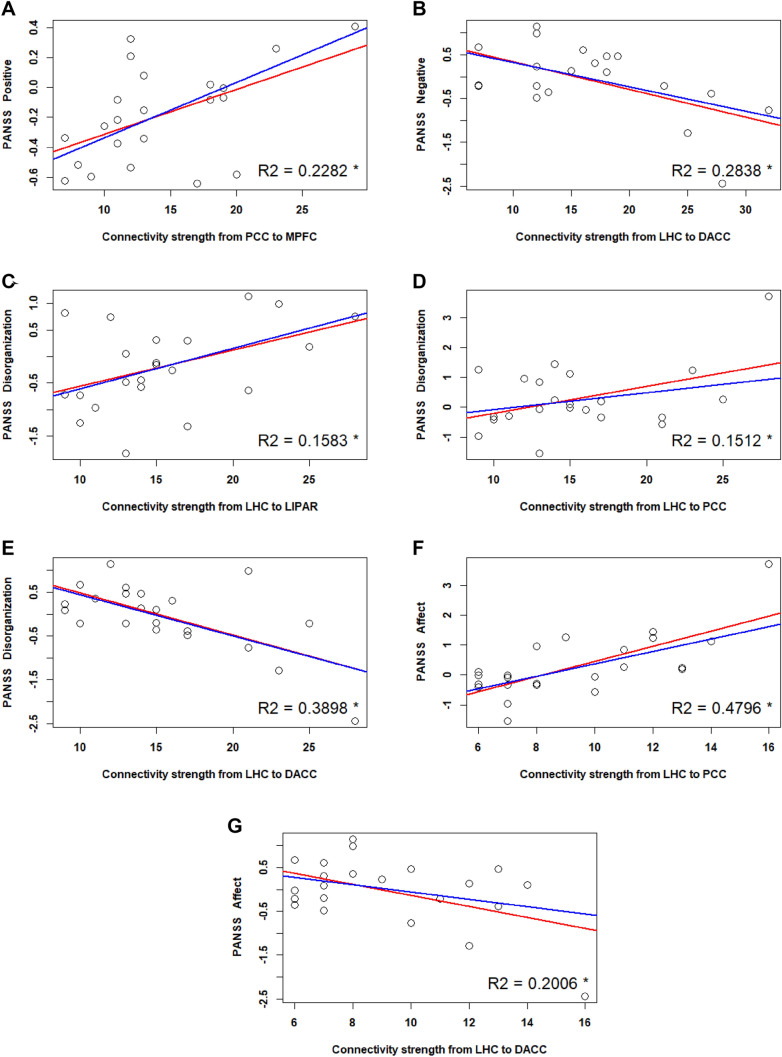
Linear regression with symptom severity (as reflected by PANSS 5 factor scores) as regressors. **A** Positive symptoms **B**: negative symptoms **C**, **D**, **E**: disorganization **F**, **G**: affect. The predicted variables are the strength of directed connectivity parameters, as mentioned on the X axis of each figure. The red regression line reflects the fit of the simple linear regression model, while the blue line reflects that of the robust regression model. The plotted R^2^ values reflect the simple linear regression model fit. The asterisk indicates an uncorrected p < 0.05 significance level

Positive symptom severity (i.e., hallucinations and delusions) correlated positively with the connection strength from the PCC to the MPFC (r = 0.44). A simple linear regression model (Fig. 7A) further showed that the relation between these variables was significant (*p* = 0.01, *p*_*Bonferroni*_ = 0.9), with a slope of 0.03 and an adjusted R^2^ value of 0.2282.

#### PANSS 5 Factors: Negative Symptoms Severity and Connectivity Strength

Negative symptom severity (i.e., social withdrawal) correlated negatively with the connection strength from the LHC to DACC (r = − 0.56). A simple linear regression model (Fig. 7B) further showed that the relation between these variables was significant (*p* = 0.009, *p*_*Bonferroni*_ = 0.13), with a slope of − 0.06 and an adjusted R^2^ value of 0.2838.

#### PANSS 5 Factors: Disorganization and Connectivity Strength

Severity of disorganized symptoms (i.e., poor attention and abstract thinking) correlated positively with the connectivity strength from the LHC to LIPAR (r = 0.45). A simple linear regression model (Fig. 7 C) further showed that the relation between these variables was significant (*p* = 0.04, *p*_*Bonferroni*_ = 0.84), with a slope of 0.03 and an adjusted R^2^ value of 0.1583.

Severity of disorganized symptoms also correlated positively with the connectivity strength from the LHC to PCC (r = 0.44). A simple linear regression model (Fig. 7D) further showed that the relation between these variables was significant (*p* = 0.04, *p*_*Bonferroni*_ = 0.83), with a slope of 0.09 and an adjusted R^2^ value of 0.1512.

It further correlated negatively with the connectivity strength from the LHC to DACC (r = − 0.56). A simple linear regression model (Fig. 7E) further showed that the relation between these variables was significant (*p* = 0.001, *p*_*Bonferroni*_ = 0.02), with a slope of − 0.09 and an adjusted R^2^ value of 0.3898.

#### PANSS 5 Factors: Affect and Connectivity Strength

Severity of affective symptoms (i.e., anxiety, depression) correlated positively with the connectivity strength from the LHC to the PCC (r = 0.71). A simple linear regression model (Fig. 7F) further showed that the relation between these variables was significant (*p* = 0.000, *p*_*Bonferroni*_ = 0.003), with a slope of − 0.25 and an adjusted R^2^ value of 0.4796.

Severity of affective symptoms also correlated negatively with the connectivity strength from the LHC to DACC (r = − 0.49). A simple linear regression model (Fig. 7 G) further showed that the relation between these variables was significant (*p* = 0.02, *p*_*Bonferroni*_ = 0.44), with a slope of − 0.12 and an adjusted R^2^ value of 0.2006.

#### PANSS 5 Factors: Resistance and Connectivity Strength

No significant correlations were found between resistance (i.e., hostility) and any directed connectivity parameters.

Finally, we also inspected the relationship between symptom severity, as reflected by individual PANSS items, and effective connectivity strength. This was achieved using a canonical correlation analysis, which in this situation was adequate given the high number of individual symptom items and connectivity parameters. This analysis was purely exploratory, and its results can be found in the supplementary material.

### Voxel Based Morphometry (VBM) Analysis of Gray Matter Volume (GMV)

We analyzed group differences of GMV for all the 13 nodes of our RSNs using an ANCOVA with total intracranial volume (TIV) as covariate. After controlling for TIV, a main effect of group remained significant for the following nodes: MPFC, LIPAR, DACC, LAI, RAI, LFEF, RFEF, LIPS. Post-hoc comparisons using Welch t-test revealed that HC had significantly higher GMV than SZ in the following nodes: MPFC, LIPAR, DACC, LAI, RAI, RFEF, LIPS. These results, as well as effects sizes are reported in Table 3.

**Table 3:** Mean TIV-adjusted gray matter volume (GMV) of each region of interest (ROI) and mean total intra-cranial volume (TIV) per group

ROI	GMV mean (SD)		HC vs. SZ (with TIV as covariate)			HC > SZ				
	HC	SZ	F(1)	*p*	ηp2	*t* *(uncorr) (Bonf. corr)*	df	*p*	*p*	*Hedge’s g*
MPFC	0.53 (0.03)	0.49 (0.05)	10.86	0.002	0.17	3.15	41	0.003	0.042	0.87
LIPAR	0.49 (0.03)	0.46 (0.05)	6.61	0.013	0.11	3.15	39	0.014	0.16	0.71
RIPAR	0.50 (0.04)	0.49 (0.05)	0.53	0.47	0.01	0.65	47	0.52	1.00	0.18
PCC	0.60 (0.06)	0.59 (0.06)	1.1	0.30	0.02	0.61	48	0.54	1.00	0.17
LHC	0.54 (0.04)	0.52 (0.04)	2.92	0.09	0.05	1.79	51	0.08	1.00	0.48
RHC	0.54 (0.03)	0.53 (0.04)	2.94	0.09	0.05	1.14	47	0.26	1.00	0.31
DACC	0.52 (0.04)	0.48 (0.04)	18.33	< 0.001	0.26	3.57	50	< 0.001	0.0014	0.95
LAI	0.56 (0.05)	0.53 (0.05)	10.16	0.002	0.16	2.23	47	0.03	0.42	0.61
RAI	0.60 (0.05)	0.56 (0.05)	12.25	< 0.001	0.19	2.80	51	0.007	0.098	0.74
LFEF	0.53 (0.07)	0.50 (0.05)	5.1	0.03	0.10	1.74	53	0.09	1.00	0.44
RFEF	0.51 (0.06)	0.47 (0.05)	14.18	< 0.001	0.21	2.95	54	0.005	0.07	0.76
LIPS	0.49 (0.07)	0.45 (0.04)	6.42	0.014	0.11	2.73	50	0.009	0.13	0.69
RIPS	0.49 (0.06)	0.47 (0.05)	2.81	0.10	0.05	1.53	54	0.13	1.00	0.40
TIV	1.59 (0.11)	1.54 (0.14)				1.51	44	0.14	1.00	0.41

Group differences in GMV were analyzed with ANCOVA using TIV as covariate. The results of post-hoc analyses with Welch two sample t-tests with both uncorrected and Bonferroni corrected p values and Hedge’s g effect sizes are summarized

#### GMV and Connectivity Strength

We additionally ran an ANCOVA analysis to check whether group differences in directed connectivity strengths remained significant when accounting for the GMV of both RSNs nodes forming each connectivity pair (listed in the column “Connection” in Table 4). Seven group differences in directed connectivity were significant after controlling for GMV: PCC to MPFC, PCC to DACC, LHC to RHC, LHC to LIPAR, RFEF to LHC, LIPS to RHC, RIPS to DACC (see Table 4). Following post-hoc Welch t tests and Bonferroni correction, these effects were no longer significant. Nevertheless, the effect sizes for most of the group differences in directed connectivity were of average magnitude (i.e. ≥ 0.5), which renders these group differences non-negligible.

**Table 4:** Group differences in mean directed connectivity strengths for each region of interest (ROI)

Connection	Connectivity strength Mean (SD)		HC vs. SZ (adjusted for GMV)			HC > SZ				
	HC	SZ	F (1)	*p*	ηp2	t	df	*P (uncorr)*	*p* *(Bonf. corr)*	*Hedge’s g*
MPFC to LHC	0.05 (0.33)	0.24 (0.47)	3.54	0.07	0.06	− 1.75	41	0.08	1.0	− 0.48
PCC to RAI	0.03 (0.37)	− 0.16 (0.47)	3.87	0.06	0.07	1.94	45	0.06	1.0	0.52
PCC to MPFC	0.03 (0.43)	− 0.18 (0.34)	4.24	0.05	0.08	2.08	54	0.05	1.0	0.54
PCC to RHC	0.04 (0.44)	− 0.19 (0.41)	3.48	0.07	0.06	1.96	53	0.06	1.0	0.52
PCC to DACC	0.14 (0.3)	− 0.11 (0.4)	7.3	0.01	0.12	2.58	43	0.01	0.24	0.71
LHC to RHC	0.5 (0.46)	0.18 (0.51)	5.01	0.03	0.09	2.11	49	0.04	0.96	0.57
LHC to LIPS	- 0.12 (0.78)	0.09 (0.81)	0.79	0.38	0.02	− 0.99	51	0.33	1.0	− 0.26
LHC to LIPAR	0.17 (0.6)	− 0.25 (0.8)	5.29	0.03	0.09	2.16	44	0.04	0.96	0.59
LHC to DACC	− 0.27 (0.44)	− 0.06 (0.74)	1	0.32	0.02	− 1.27	37	0.21	1.0	− 0.36
LHC to RIPS	− 0.001 (0.57)	0.27 (0.7)	2.64	0.11	0.05	− 1.59	46	0.12	1.0	− 0.43
LHC to RIPAR	0.28 (0.79)	− 0.04 (0.05)	1.9	0.17	0.04	1.59	54	0.12	1.0	0.41
LHC to RAI	− 0.44 (0.54)	0.33 (0.9)	3.11	0.08	0.06	− 1.84	38	0.08	1.0	− 0.51
LHC to PCC	0.45 (0.66)	0.22 (1.03)	0.88	0.35	0.02	0.95	39	0.35	1.0	0.26
LHC to LFEF	− 0.03 (0.56)	0.2 (0.53)	2.76	0.1	0.05	− 1.6	53	0.12	1.0	− 0.42
RHC to RAI	− 0.13 (0.85)	0.08 (0.94)	1.04	0.31	0	− 0.87	49	0.39	1.0	− 0.23
RHC to RFEF	0.08 (0.55)	0.24 (0.44)	2.64	0.11	0.01	− 1.23	54	0.23	1.0	− 0.32
RFEF to LHC	0.14 (0.38)	− 0.05 (0.33)	3.57	0.07	0.06	2.03	54	0.05	1.0	0.53
RFEF to RIPS	0.33 (0.41)	0.11 (0.47)	2.68	0.11	0.05	1.91	49	0.06	1.0	0.51
RHC to LHC	0.45 (0.5)	0.22 (0.55)	3.55	0.07	0.06	1.61	50	0.12	1.0	0.43
RHC to RIPS	− 0.03 (0.93)	0.3 (0.64)	2.03	0.16	0.04	− 1.53	53	0.13	1.0	− 0.39
RHC to LAI	− 0.3 (1.21)	0.08 (0.81)	2.16	0.15	0.04	− 1.41	52	0.17	1.0	− 0.36
LIPS to RHC	0.07 (0.38)	− 0.18 (0.37)	5.57	0.02	0.1	2.44	52	0.02	0.48	0.65
RIPS to DACC	0.04 (0.33)	− 0.19 (0.41)	5.2	0.03	0.1	2.23	46	0.03	0.72	0.60
RAI to LHC	0.16 (0.4)	− 0.02 (0.36)	4.03	0.05	0.07	1.82	53	0.08	1.0	0.48

Welch two samples *t* tests with both uncorrected and Bonferroni corrected p values and Hedge’s g effect sizes are summarized. The arrows indicate the connectivity direction between nodes

#### Effects of Illness Duration and Medication on Directed Connectivity Strength in SZ

Finally, we also checked whether illness duration and medication modulated connectivity strength in SZ. First, we ran two additional ANCOVA analyses, one with illness duration and another one with medication (i.e., mg/day of chlorpromazine equivalent) as covariates. In order to adequately code each patient’s medication regimen, we used the Chlorpromazine equivalent according to the method proposed by Gardner et al. (2010). Illness duration did not significantly modulate any of the connectivity strengths (i.e., p > 0.15, η^2^ < 0.09). Medication did however significantly modulate the connectivity strengths from LHC to RHC (*p* < 0.001, η^2^ = 0.4), from RHC to RAI (*p* = 0.035, η^2^ = 0.18), from RFEF to RIPS (*p* = 0.001, η^2^ = 0.38), and from RAI to LHC (*p* = 0.007, η^2^ = 0.28). Next, we ran Pearson correlation analyses between medication and connectivity strength in the patient group. Significant negative medium-sized correlations were found between medication and the following four connectivity pairs; LHC to RHC (r = − 0.64, *p* < 0.001), RHC to RAI (r = − 0.42, *p* = 0.04), RFEF to RIPS (r = − 0.62, *p* = 0.001) and RAI to LHC (r = − 0.52, *p* = 0.007).

## Discussion

In this study, we investigated group differences in effective connectivity between HC and SZ with respect to brain areas which are known to play a role in three major RSNs: DMN, SAN and DAN. Of particular interest to us was the bilateral hippocampus (i.e., LHC and RHC), which have been found to show distinctive connectivity patterns as well as volumetric alterations in SZ. We also explored the link between GMV and directed connectivity by checking whether the strength of directed connectivity in SZ persisted when controlling for GMV of node pairs. Additionally, we also checked whether symptom severity could predict connection strengths. Finally, we assessed whether hierarchical strengths of and between the three RSNs revealed any group differences. Our results point to reliable group differences which: (1) consolidate the role of the hippocampus as bearing key disease-specific alterations in SZ, and (2) narrow the pool of potential biomarkers which can serve as clinically valuable indicators of disease severity and progression.

The dysconnection hypothesis posits that SZ symptoms arise from impaired brain network function, and not (only) from discrete structural and/or functional alterations (Friston and Frith 1995). Since a network is characterized by directed interactions between brain areas, effective connectivity methods, especially DCM, appear to be preferable to the undirected (i.e., functional) ones. These directed approaches have demonstrated increased reliability (e.g., Schuyler et al. 2010) and can point to potential *intermediate phenotypes* (i.e., markers of heritability) in SZ (Cao et al. 2016). Our current results largely speak in favor of the dysconnection hypothesis. We found no less than 24 distinct patterns of directed connectivity which were significantly different between HC and SZ, most of these originating in the PCC, LHC and RHC (i.e., nodes belonging to the DMN). In SZ, the connections from the PCC to other DMN and SAN nodes were significantly weaker in comparison to HC, but only the PCC to DACC connection strength group difference remained significant when controlling for the GMV of both nodes. Additionally, the connections from the LHC to other DMN nodes were significantly weaker in SZ than in HC, while the connections from the LHC to the SAN and DAN nodes were significantly stronger in SZ than in HC. Finally, the connections from the RHC to other nodes of the SAN and DAN were also significantly stronger than in HC, but weaker towards other DMN nodes. After controlling for the GMV of both node pairs however, only the connection strengths from the LHC to RHC and to LIPAR remained significantly higher in HC. Overall, these results point to a general pattern of exacerbated connectivity strength from the nodes of the DMN to those of the SAN and DAN in the SZ group.

To our knowledge, this is the first paper which investigated hierarchical strength differences of large scale RSNs between SZ and HC. Though directed connectivity between the hippocampus and other brain areas of SZ has been previously investigated by Zhou et al. (2018b), it was done on data collected from a task-based fMRI memory paradigm and not on resting state data. We have however chosen to investigate group differences in directed connectivity between these three networks during rest. The particular appeal of using rsfMRI data is due to it being more easily acquired from a wide variety of patients, including some who might be more severely impaired and who might not otherwise be suitable for participating in task-based paradigms.

Previously, Zhou et al. (2018a) have revealed that a hierarchical relation between the nodes of these three networks occurs in the healthy general population, with nodes of the DMN exciting those of the SAN and the DAN. Our results suggest that even though this hierarchy is maintained in SZ, the strength of connections within this hierarchy are significantly increased compared to HC. As opposed to Zhou et al. (2018a) however, we were unable to replicate this hierarchical relationship in our HC group; on the contrary, in our HC sample, the DMN exited both the DAN and the SAN. One important distinction between their study and ours which might have led to this divergent finding is that Zhou et al. considered the core DMN only, without including the hippocampus. For our study, however, given our special interest in hippocampal dysconnectivity, we preferred the extended DMN conceptualization. A second distinction is that these authors included additional areas in their SAN (i.e., bilateral anterior prefrontal cortices) and DAN (i.e., bilateral inferior frontal gyri). A complete replication of their results would therefore be dependent on the initial definition of the respective RSNs. Finally, differences regarding the sample characteristics, such as the age and sample size of our HC group, between our study and the one of Zhou et al. (2018a) could have also played a role in the discrepancies between our results and theirs.

A growing body of research has acknowledged and tackled the heterogeneity of SZ (e.g., Schnack 2019) in recent years. In line with this trend, we also aimed to quantify the heterogeneity of our sample by analyzing the relationship between symptom severity and directed connectivity. We found that both positive and negative symptom strength significantly predicted directed connections originating in the hippocampus, namely: negative symptoms negatively predicted the directed connectivity from the LHC to DACC and RHC, and positively predicted that from the LHC to LIPAR, while positive symptoms negatively predicted the directed connectivity from the RHC to LHC and negatively predicted that from the LHC to LIPAR. We therefore show that hippocampal connections to nodes of the SAN can be predicted by the severity of negative symptoms. In addition to previous research (e.g. Duan et al. 2015; Garrity et al. 2007) which found exclusively predictive relationships between connectivity strengths and positive symptom severity, we found that negative symptom severity was also a significant predictor. A potential explanation for this may reside in our choice of a DCM-based directed connectivity assessment, i.e., using symptom severity to predict the strength of directed connections between two nodes rather than the time courses of each single node. We expanded this analysis by further analyzing the relationship between additionally computed PANSS 5 factor subscales and connectivity strength. In six out of seven cases, symptom severity significantly predicted connection strengths originating in the LHC, further supporting the fact that hippocampus dysconnectivity is tightly related to disease severity. Finally, this conclusion is further supported by the additional canonical correlation analysis summarized in the supplementary material.

Although previous authors (e.g., Radulescu et al. 2014) have reported reduced hippocampal volumes in SZ compared to HC, we found no significant hippocampal GMV group differences in our sample. Nevertheless, total hippocampal GMV group differences have not always been replicated, which led some authors to recommend a sub-hippocampal structures approach instead (e.g., Folley et al. 2010), or multimodal methodologies (e.g., Boyer et al. 2007). We did however find significant group differences in GMV of other nodes, such as: MPFC, LIPAR, DACC, LAI, RAI, RFEF and LIPS, though only DACC survived the Bonferroni correction in post-hoc testing. For this reason, we re-checked the connectivity group differences by controlling for GMV. Seven group differences in directed connectivity remained significant even after controlling for GMV: PCC to MPFC, PCC to DACC, LHC to RHC, LHC to LIPAR, RFEF to LHC, LIPS to RHC, RIPS to DACC, but none of these subsequently survived Bonferroni corrections in post-hoc testing. Despite this, the effect sizes of group differences were larger than 0.5, therefore rendering them non-negligible. This is a novel finding, considering we investigated the modulating effect of GMV on directed connection strengths. Previous findings did however point to a relationship between GMV and functional connectivity in SZ (e.g., Zhang et al. 2015) and HC (Müller et al. 2015).

Finally, we checked whether illness duration and medication could have acted as confounded variables. Illness duration did not significantly modulate group differences in directed connectivity strength. However, medication did modulate the connectivity strengths from the LHC to the RHC, RHC to RAI, RFEF to RIPS, and RAI to LHC. It is not implausible that antipsychotic medication can alter brain connectivity, as it has been previously shown that functional connectivity involving the hippocampus, anterior insula and other frontal areas increase as a result of symptom reduction following pharmacological interventions in SZ (e.g., Sarpal et al. 2015; Kraguljac et al. 2016). Moreover, it has also been shown that antipsychotic medication increases the directed connectivity strength from the hippocampus to frontal regions (Hutcheson et al. 2014). It is therefore not completely clear whether group differences in these directed connections could be found with non-medicated samples, so the group differences that we found in these connectivity pairs could have been in fact confounded by medication. Additionally, the fact that antipsychotic medication appeared to alter these connections could also point to potential sites for assessing pharmacological effects.

The main limitation of the current study resides in its small sample size. A suitable follow-up aimed at overcoming this drawback would therefore be the replication of our directed connectivity analysis on a larger sample, such as The Center for Biomedical Research Excellence (COBRE). An additional limitation is that the number of resting state scans in our study was relatively low (Razi et al. 2015). Nevertheless, previous DCM analyses have been successful despite a similarly reduced number of time points (e.g., 180 in Zhou et al. 2018a, and 150 in Almgren et al. 2018). We would add that especially when clinical samples are concerned, oftentimes it is extremely difficult to acquire (good quality) data using lengthy fMRI protocols (hence the special appeal of rsfMRI). Finally, another limitation is that while we ensured that our SZ sample were within normal range with respect to their cognitive abilities, we did not pursue this aspect in detail, as investigating the relationship between cognitive abilities and RSNs was beyond the scope of our study.

In conclusion, our results support a robust relation between hippocampal dysconnectivity and symptom severity in SZ. Moreover, we trust that our approach, based on exploring directed dysconnectivity characteristics of large-scale RSNs and their relation to symptoms and structure, can offer valuable insight into putative mechanisms of disease and intervention.

## Declaration

## Conflict of interest

None of the authors have any conflict of interests to declare.

## Supplementary Information

Below is the link to the electronic supplementary material.

Supplementary file1 (DOCX 18 kb)

## Data Availability

The data-set can be shared upon request by contacting the last author.
