# Supplementary material for: Effective Connectivity of the Hippocampus Can Differentiate Patients with Schizophrenia from Healthy Controls: A Spectral DCM Approach
Source: Brain Topogr. 2021 Sep 4;34(6):762–78. doi: 10.1007/s10548-021-00868-8 (PMC8556208; doi:10.1007/s10548-021-00868-8)
Supplement: Supplementary file 1 — Supplementary file1 (DOCX 18 kb) [file 10548_2021_868_MOESM1_ESM.docx]

**6. Supplementary material

Canonical correlation**Before the canonical correlation analysis (CCA) could be performed, it was necessary to examine and eliminate the collinearity within the two sets of data, i.e., symptom items and connectivity parameters. A variance inflation factor larger than 5, or a Spearman’s rho larger than .6 were taken as criteria for the elimination of the variables driving those values. The canonical correlation analysis was run using the *cancor* function from the R base, as well as the functionality implemented in the *CCP* package. The resulting variables which were included in the canonical correlation analysis were:

(1) Symptoms
- p02: Conceptual disorganisation;
- p03: Hallucinatory behaviour;
- p04: Excitement;
- p05: Grandiosity;
- n03: Poor rapport;
- n05: Difficulty in abstract thinking;
- n06: Lack of spontaneity and flow of conversation;
- n07: Stereotyped thinking.

(2) Connectivity parameters: MPFC to LHC, PCC to MPFC, PCC to RAI, LHC to DACC, LHC to LFEF, RHC to LHC, RHC to RFEF, RAI to LHC, RFEF to LHC, RFEF to RIPS, LIPS to RHC.

Only one CCA mode relating symptom severity to effective connectivity strength was highly significant, also indicated by a very small Wilk’s Lambda value (Wilk’s Lambda = 5.2 * 10^-20^, P<10^-8^ ). Next, we explored standardized canonical coefficients of this first CCA mode. The symptoms with the highest loads on the first canonical dimension are: p02 (with -0.26) and n06 (with 0.16). The connectivity parameters with the highest loads on the first canonical dimension are RHC to LHC (with 0.15) and RFEF to RIPS (with 0.13). These values are given in Table 5 below:

**Table 5**. Summary of all items considered and finally included in the CCA analysis, together with their standardized canonical loads. The loads in bolded font are the highest ones.

| **PANSS items** | | **Included in CCA** | | | **Load value** |
| --- | --- | --- | --- | --- | --- |
| p01 Delusions | |  | | |  |
| p02 Conceptual disorganisation | | ✓ | **-0.260207696** | | |
| p03 Hallucinatory behaviour | | ✓ | | -0.064723702 | |
| p04 Excitement | | ✓ | | 0.023327034 | |
| p05 Grandiosity | | ✓ | | -0.007352677 | |
| p06 Suspiciousness/persecution | |  | |  | |
| p07 Hostility | |  | |  | |
| n01 Blunted affect | |  | |  | |
| n02 Emotional withdrawal | |  | |  | |
| n03 Poor rapport | | ✓ | | -0.059030531 | |
| n04 Passive/apathetic social withdrawal |  | | |  | |
| n05 Difficulty in abstract thinking | | ✓ | | 0.005624570 | |
| n06 Lack of spontaneity & flow of conversation | | ✓ | | **0.162312927** | |
| n07 Stereotyped thinking | | ✓ | | 0.035591232 | |
| **Connectivity parameters** | |  | |  | |
| MPFC to LHC | | ✓ | | 0.040213940 | |
| PCC to MPFC | | ✓ | | -0.072073515 | |
| PCC to RHC | |  | |  | |
| PCC to DACC | |  | |  | |
| PCC to RAI | | ✓ | | 0.091180330 | |
| LHC to LIPAR | |  | |  | |
| LHC to RIPAR | |  | |  | |
| LHC to PCC | |  | |  | |
| LHC to RHC | |  | |  | |
| LHC to DACC | | ✓ | | 0.074057983 | |
| LHC to RAI | |  | |  | |
| LHC to LFEF | | ✓ | | -0.002082179 | |
| LHC to LIPS | |  | |  | |
| LHC to RIPS | |  | |  | |
| RHC to LHC | | ✓ | | **0.152097151** | |
| RHC to LAI | |  | |  | |
| RHC to RAI | |  | |  | |
| RHC to RFEF | | ✓ | | 0.052565514 | |
| RHC to RIPS | |  | |  | |
| RAI to LHC | | ✓ | | 0.088745969 | |
| RFEF to LHC | | ✓ | | 0.040846441 | |
| RFEF to RIPS | | ✓ | | **0.131711507** | |
| LIPS to RHC | | ✓ | | -0.090056366 | |
| RIPS to DACC | |  | |  | |
